# Ocular Neuromyotonia—A Rare but Reversible Cause of Intermittent Diplopia

**DOI:** 10.1002/mdc3.70103

**Published:** 2025-04-21

**Authors:** Parthvi Ravat, Paul Stockle, Mahadya Rahman, Pegah Ahmadiyan Ardestani, Andrew Lee

**Affiliations:** ^1^ Centre for Neuroscience Innovation Flinders University Adelaide South Australia Australia; ^2^ Flinders University Adelaide South Australia Australia

**Keywords:** bedside diagnosis, demyelination, diplopia

Ocular NeuroMyotonia (ONM) is a medically treatable cause of episodic diplopia, which may develop spontaneously due to a tonic contraction or spasms of the extraocular muscles. Here, we present one such case that emphasizes the learning that this entity might be detected on clinical examination, especially when looked for.^1^

A 39‐year‐old female presented with intermittent double vision when looking to the right, followed by spontaneous resolution within 60 to 90 seconds. She had a history of radiotherapy for right ear cancer when she was 8 years old. In the clinic, she had a normal ophthalmological and neurological examination except for a long‐standing mild ptosis in the right eye, which she noticed in her twenties. However, it has not bothered her; it has been static and possibly due to levator aponeurosis dehiscence. There was no fatigability, myasthenic eyelid signs were absent, and thyroid function tests were normal. As the diplopia was random and intermittent, we could not elicit diplopia on looking to the right in the clinic. Furthermore, no oscillopsia, vertical deviation, or nystagmus was noted. Normal ocular alignment was measured by an alternate cover test both at a distance and near, which made decompensating heterotopia less likely.^1^ However, extended gaze deviation and examination helped us clinch the diagnosis. The abducens nerve was solely affected, leading to intermittent episodic tonic exotropia on lateral gaze to the affected side after a latent period of a few seconds. This led to restricted adduction lasting for a few seconds (See Video 1).

**VIDEO 1:** The video initially shows the patient depicting complete extra‐ocular eye movements in horizontal and vertical directions. She has a baseline mild right eye ptosis, possibly secondary to levator aponeurosis dehiscence. Thereafter, she goes on to look to her left without any abnormality. However, subsequently, as she looks to her right, one can notice a sustained eye deviation to the right only in her right eye. She says that this is no longer under her control. After a few seconds, the right eye returns to the baseline position. This abnormal tonic right eye movement seen here is ocular neuromyotonia.

This is termed as ONM, described in the early 1970s, where a parallel is drawn to myotonia in the peripheral nerves.^2^ The commonly proposed mechanism of neuromyotonia is hyperexcitability secondary to segmental myelin damage and dysfunction of potassium channels,^3^ which leads to repetitive motor unit discharge.^4^, ^5^ In our case, the insulting event is hypothesized to be radiation‐induced microangiopathy of the cranial nerves, thus leading to impaired ephaptic transmission.

ONM is found more commonly in females, with the abducens nerve (lateral rectus) being the most affected muscle, followed by the oculomotor nerve.^2^, ^5^ The most common cause is a delayed side effect of oncological irradiation and is usually seen within a few years of radiation therapy.^3^ However, some differentials must be actively ruled out, such as cranial nerve palsies, tumor relapses, other tumors noted with this rare complication such as meningioma, pontomedullary venous angioma, intramedullary vascular lesion, clivus tumor, pituitary adenoma, chondrosarcoma, rhabdosarcoma, a thalamic glioma, cerebellar ependymoma.^2^, ^5^ Rare causes also include neurovascular conflict, such as a cranial nerve getting entwined around an artery or a dolichoectasia or aneurysm of the internal carotid artery.

Diplopia might not be elicited every time or might have a latent period before the myotonia begins. Videooculography, electromyography (which in such cases is expected to show sustained motor unit discharge during periods of expected muscle relaxation), and muscle biopsy might help.

Treatment includes trying a lottery of membrane stabilizers such as gabapentin, carbamazepine, lacosamide, etc. She demonstrated no response to Gabapentin. However, Carbamazepine reduced her tonic pulses of exotropia in duration and frequency. She could see things in lateral gaze without a myotonic response.

## Author Roles

(1) Research project: A. Conception, B. Organization, C. Execution; (2) Statistical Analysis: A. Design, B. Execution, C. Review and Critique; (3) Manuscript: A. Writing of the first draft, B. Review and Critique.

P.R.: 1A, 1C, 2A, 3A, 3B.

P.S.: 1C, 3B.

M.R.: 1C, 3B.

P.A.A.: 1C, 3B.

A.L.: 1A, B, 3B.

## Disclosure

**Ethical Compliance Statement:** Ethical guidelines were followed in the absence of an IRB including complete informed written and verbal consent was taken from the patient. The recording of the video was done in the clinical settings only after ensuring complete voluntary participation from the patient. Written and verbal Informed patient consent was obtained from the patient and stored in her electronic medical record. All authors confirm that we have read the Journal's position on issues involved in ethical publication and affirm that this work is consistent with those guidelines.

**Funding Sources and Conflicts of Interest:** No specific funding was received for this work, and the authors declare that there are no relevant conflicts of interest.

**Financial Disclosures for the Previous 12 Months:** The authors declare that there are no additional disclosures to report.

## Data Availability

The data that supports the findings of this study are available in the supplementary material of this article.
